# Notes and News

**Published:** 1898-12-17

**Authors:** 


### Notes and News.

(Collected and Communicated.)

The London County Council is to be congratulated on having been rescued from the absurdity of paying a higher salary to the manager of its tramways than it does to the head of its Public Health Department.

The Oxford University Press is publishing a monograph by Professor Veley, F.R.S., and Lilian J. Veley (*née* Gould), on "The Micro-organism of Faulty Rum." This should form a subject of considerable interest, as showing how wide is the scope of bacteriology. One would hardly look upon strong spirit as a likely medium for the growth of micro-organisms.

Dust is a subject engaging public attention in Melbourne. It is difficult for any English person to understand how the whole of Australia, towns and country, suffers from dust. Dust storms occasionally happen, but dust that insinuates itself into hair, ears, and nose, and every corner of house, shop, or warehouse, is perennial. So far no effort has been made to cope with the fiend in Melbourne, except by occasional watering of the streets, in a way that leaves them, from the peculiarity of the soil, worse than ever when dry. The illness provoked by dust, and the amount of goods damaged by dust are both large. The city Corporation have meantime consented to purchase more water-carts.

On Tuesday a large scheme was inaugurated by the City of London which will very much widen the area dependent upon the Foreign Cattle Market at Deptford for its meat supply. The scheme involves a considerable extension of the appliances for slaughtering and for storing meat, as well as the construction of a branch line by which the market will be put in connection with the general railway system of the country. The responsibility of the City of London in regard to the inspection of imported meat thus becomes very great. It becomes, indeed, a matter of almost national concern; and it is to be hoped that the committee in charge of this department will, at last, rise to the occasion, and will inaugurate a complete and scientific examination of all the meat which passes through its hands in place of the very haphazard proceedings which are at present in vogue.

The question of cremation has never aroused any great public interest in Victoria, but it is kept alive by a cremation society formed of Mr. Justice à Becket, Mr. H. K. Rusden (Parliamentary Librarian), Dr. Gresswell (chairman of the Board of Public Health), Mr. Theyre Weigall (formerly Curator of Intestate Estates), and a few others. The society wishes to erect a crematorium, but subscriptions come in very slowly. None of the trustees of the cemeteries will erect one, and wherever it is proposed to build one a cry is sent up by the residents regarding the damage it will be to their property. At the meeting of the Cremation Society, on September 22nd, Dr. Gresswell agreed to take steps towards arranging for a deputation of the medical profession to wait upon the Minister of Public Health in regard to the question.

In our Christmas Plate this year we offer to our readers two simple sketches illustrating two phases of life at Christmas time—life in the hospital and life in the home. They are but sketches; the details are left to be filled up according to the knowledge and experience of each reader, but the feeling which they suggest to us is that of the broad contrast which exists between a Christmas as it is in the hospital and as it is experienced in far too many a home. At no time of the year is poverty more distressing than at Christmas. When work is slack, when all who are better off are enjoying themselves, and when in consequence trade is at a standstill, the poor find life the hardest, besides feeling the bitterness of the contrast with those whose circumstances are more conducive to enjoyment. The contrast is, indeed, a marked one; on the one hand we have hunger, the bare cottage, the bare board, and general wretchedness; on the other, there is plenty and comfort, and, more valuable still, there is sympathy. In the one sketch we see each sufferer suffering by herself; in the other, the little child receives both food and love.

The Report of the Medical Officer to the Local Government Board, which we have just received, contains much that is of great interest besides the usual reports on the various outbreaks of infectious disease which have occurred. We would especially direct attention to what is said by Sir Richard Thorne in regard to vaccination. He says: "The number of children now born in England and Wales who in one way or another escape vaccination is probably not much less than one-third of the whole. In this way the country is being prepared for widespread epidemics of small-pox such as have been unknown to the present generation, unless, indeed, the invariable rush and clamour for immediate vaccination on the part of those who have neglected or declaimed against the operation during times of freedom from smallpox should be capable of being so far met in the moment of emergency as largely to mitigate the impending disaster. It will be observed from the returns for 1895 that 2,962 children were registered as insusceptible of vaccination. In this connection I would point out that the number of consecutive primary vaccinations by the Board's own officers without the occurrence of a single instance of so-called insusceptibility now reaches 107,180." We cannot but feel that many certificates of insusceptibility are granted on grounds and from motives which will not bear investigation.

